# Expression Pattern of iNOS, BCL-2 and MMP-9 in the Hip Synovium Tissue of Patients with Osteoarthritis

**DOI:** 10.3390/ijms22031489

**Published:** 2021-02-02

**Authors:** Davor Caric, Sandra Zekic Tomas, Natalija Filipovic, Violeta Soljic, Benjamin Benzon, Sandro Glumac, Ivan Rakovac, Katarina Vukojevic

**Affiliations:** 1 Department of Orthopaedics and Traumatology, University Hospital in Split, Spinciceva 1, 21000 Split, Croatia; caric.davor@gmail.com; 2 Department of Pathology, Forensic Medicine and Cytology, University Hospital in Split, Spinciceva 1, 21000 Split, Croatia; szekic@mefst.hr; 3 Department of Anatomy, Histology and Embryology, School of Medicine, University of Split, Soltanska 2, 21000 Split, Croatia; natalija.filipovic@mefst.hr (N.F.); benjamin.benzon@mefst.hr (B.B.); 4 Department of Histology and Embryology, School of Medicine, University of Mostar, Kralja Petra Kresimira IV, 88000 Mostar, Bosnia and Herzegovina; violeta.soljic@mef.sum.ba; 5 Department of Anesthesiology and Intensive Care, University Hospital in Split, Spinciceva 1, 21000 Split, Croatia; sandro.glumac@gmail.com; 6 Department of Natural and Health Sciences, Juraj Dobrila University of Pula, Pula, Zagrebačka ul. 30, 52100 Pula, Croatia; ivan.rakovac@gmail.com

**Keywords:** osteoarthritis, Hip, iNOS, BCL-2, MMP-9, cartilage, subchondral tissue, synovia

## Abstract

Hip osteoarthritis (HOA) is characterized by degradation of the cartilage and synovitis. However, the pathohistological effects of synovial tissue inflammation on HOA are not clear. The aim of this study was to evaluate the expression of iNOS, BCL-2 and MMP-9 markers in different synovial cell populations. A total of 32 patients were evaluated retrospectively. Age, sex, height, weight, body mass index were recorded and lymphocyte, fibrocytes and macrophages were analysed in tissue sections. Osteoarthritis cartilage histopathology assessment system (OARSI), Western Ontario and McMaster Universities Osteoarthritis Index (WOMAC), Krenn score, Harris Hip Score (HHS) and Kellgren–Lawrence (K-L) grading of the hip joints were performed. Total hip arthroplasty was performed on 32 patients and controls. Patients were divided into two groups according to their disease severity. The tissues were immunohistochemically analysed. K-L grade and Krenn score differ between all three groups, but also between moderate and severe OA. Synovial lining cell layer, resident cells in stroma and especially inflammatory infiltration were increasing with severity of OA. iNOS expression in both intima and subintima was positively correlated with Krenn score in moderate and severe osteoarthritis (OA) groups. Expression of BCL-2 in intima of severe OA patients was positively correlated with Krenn score. In conclusion, iNOS, BCL-2 and MMP-9 are involved in the regulation of HOA. Our study indicates a relationship between the pathohistological features, the synovial inflammation and the cartilage condition at the time of hip replacement due to OA or femoral neck fracture.

## 1. Introduction

Osteoarthritis is a degenerative joint disease that causes progressive damage to articular cartilage and surrounding structures and is a common cause of pain and disability among older adults [1,2,3]. Among all osteoarthritis, hip osteoarthritis (HOA) has a huge impact on quality of life as well as significant negative economic impact due to reduced work capacity of the working population and treatment costs [4,5]. Prevalence of symptomatic HOA is 9.2% in the population older than 45 years [6]. Osteoarthritis OA is defined as a degenerative joint disorder affecting not only a cartilage and subchondral bone but whole joint including ligaments, menisci and synovial membrane and leads to cartilage degradation, inflammation of the synovial membrane, subchondral bone sclerosation, degeneration of ligaments and menisci and osteophytes formation [1,2]. Primarily the process appears to take place in the cartilage due to disturbed homeostasis of the extracellular matrix (ECM) with increase in water content, decrease in proteoglycans content of the ECM and changes in collagen type II production [7,8]. An inflammatory process or damage after trauma increases enzymatic activity and activates macrophages whose breakdown products affects chondrocytes by secreting proteolytic enzymes [9,10]. Macrophages, predominantly from the synovial membrane, phagocytize degradation products of collagen and proteoglycans causing production and release of proinflammatory cytokines: interleukin (IL)-1, IL-2, IL-6, IL-12, TNF-α and cyclooxygenase-2 (COX-2) [9,10]. Cytokines bind to chondrocyte receptors which is followed by increased synthesis and release of matrix metalloproteinases (MMPs) and change in production of collagen type II [11]. The synovial membrane in HOA shows thickening, increased vascularization and monocyte and lymphocyte infiltration [12]. The synovial membrane is composed of two layers: intima and subintima [13] and contains macrophages, Fibroblast Like Synoviocytes (FLS), endothelium of blood vessels, smooth muscles, lymphocytes and plasma cells [14,15,16]. Cytokines, adipokines, MMPs, COX-2, Inducible Nitric Oxide Synthase (iNOS) are secreted by synovial cells, as well as chondrocytes, so the synovial membrane has an important role in OA pathogenesis [17,18,19,20]. Considering presence of pro-inflammatory factors in synovial fluid and histological changes occurring predominantly in the synovial membrane, it has been proposed that OA is an inflammatory disease [21]. In the last few years there has been growing evidence that the FLS mediate inflammatory synovitis in OA by producing cytokines, NO and prostaglandin E_2_ [22]. In OA, cartilage and the synovial membrane produce large quantities of nitric oxide (NO) [19]. NO in OA mediates the expression of inflammatory factors, inhibits synthesis of collagen and proteoglycan synthesis, and induces chondrocyte apoptosis and pain [23,24]. MMPs are the family of enzymes involved in the ECM breakdown [25]. MMPs are produced by both synoviocytes and chondrocytes and their activation causes irreversible tissue destruction, targeting collagens (types II, IX, and XI) and proteoglycans [25,26] as main components of the ECM. Contrary to inflammation, antiapoptotic factors maintain tissue homeostasis through the anti-apoptotic BCL-2 protein that controls mitochondrial apoptotic signaling by preventing mitochondrial permeabilization and release of cytochrome c [27]. Fibroblast-like synoviocytes survival is dependent of apoptosis and over-expression of BCL-2 protects them from programmed cell death [28].

To our knowledge, there are no studies that analyze distribution patterns of iNOS, MMP-9 and BCL-2 in the synovial membrane of severe HOA. The aim of the study was to establish a correlation between of iNOS, BCL-2 and MMP-9 synovial membrane distribution patterns and radiological grades, histological grades and patient clinical scores as well. The differences in the expression pattern of these markers in different synovial membrane cell populations could be the target of a new pharmacologic agent that can relieve the symptoms of HOA.

## 2. Results

**Figure 1:**
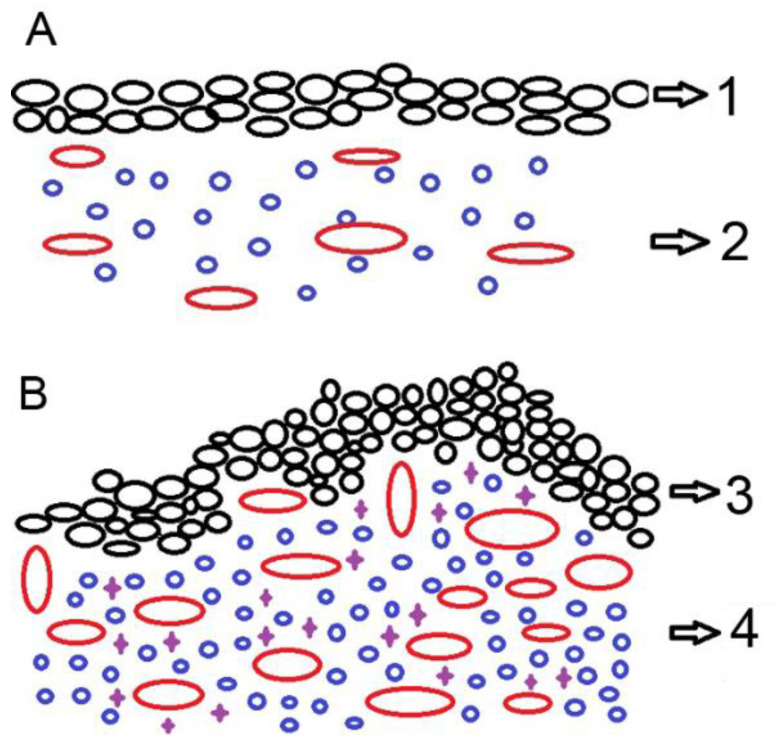
Schematic drawing of synovial membrane of patients with moderate (**A**), and severe (**B**) hip osteoarthritis (HOA). The epithelial lining (black circles) represents intima and stroma underneath represent subintima; 1—synovial surface lined by moderate hyperplasia of synovial cells (black circles); 2—underlying stroma with moderate amount of lymphocytes (blue circles) and blood vessel proliferation (red circles); 3—severe hyperplasia of synovial cells (black circles) in a papillary pattern; 4—underlying stroma with abundant lymphocytes (blue circles), macrophages (violet crosses) and pronounced blood vessel proliferation (red circles).

**Figure 2:**
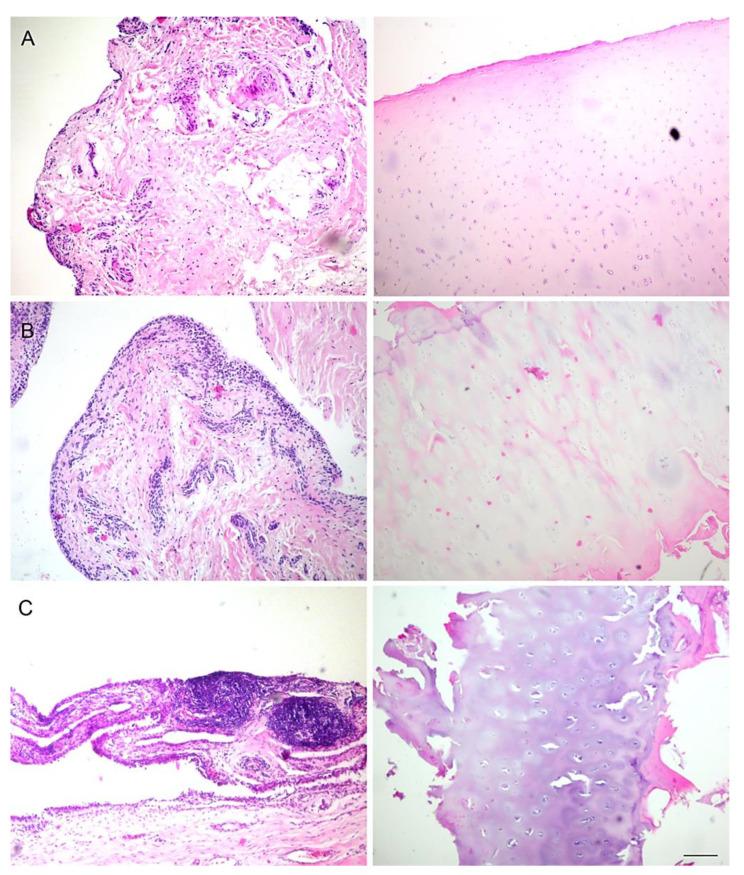
Synovial membrane (first column) and cartilage (second column) of control patients (**A**), and patients with moderate (**B**) and severe (**C**) hip osteoarthritis (HOA). Hematoxylin and Eosin staining. Magnification ×40, scale bar = 40 µm.

In total there were 32 patients (9 men; 23 women). There was no statistical difference between groups according to age and BMI (*p* = 0.884 and *p* = 0.055, respectively) (Table 1). Symptoms average duration was 2.5 years in moderate OA and 3 years in severe OA. Although the osteoarthritis cartilage histopathology assessment system (OARSI) score differs significantly between all three groups, when we compare only moderate and severe OA, there are no significant differences (Figure 1 and Figure 2, Table 1). Similarly, the Harris Hip Score (HHS), visual analogue scale (VAS) and The Western Ontario and McMaster Universities Osteoarthritis Index (WOMAC) score showed no difference as well. On the other hand, the Kellgren–Lawrence (K-L) grade and Krenn score differ between all three groups, but also between moderate and severe OA (Table 1).

Synovial lining cell layer, resident cells in the stroma and especially inflammatory infiltration increased with severity of OA. Additionally, lymphoid aggregates in the synovium were not seen in normal tissue and only rarely in moderate OA but were present in a quarter of severe OA synovial membranes.

**Figure 3:**
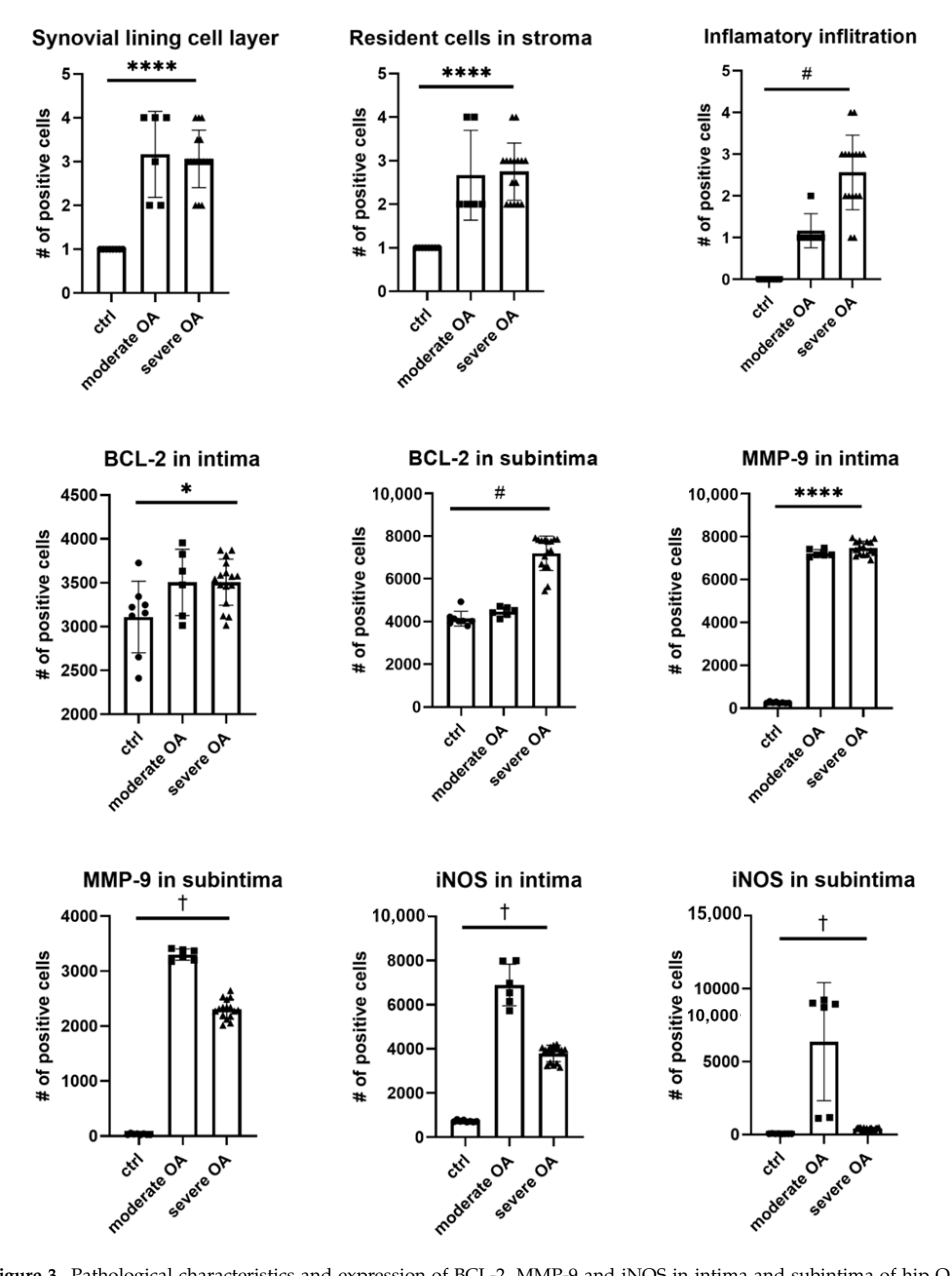
Pathological characteristics and expression of BCL-2, MMP-9 and iNOS in intima and subintima of hip OA patients. Legend: ctrl (control), osteoarthritis (OA); * *p* < 0.05, ****, *p* < 0.0001 for ANOVA; #, *p* < 0.05 for the test for linear trend; † *p* < 0.05 for the test for parabolic (quadratic) trend.

iNOS expression in the intima showed a parabolic trend with the highest level of 6899 ± 940.8 positive cells/mm^2^ in moderate OA (Figure 3). Similar results were noted in subintima. BCL-2 expression was similar in intima of moderate and severe OA, however when compared to control, BCL-2 expression was higher in the latter groups. However, BCL-2 expression in subintima showed a linear trend of increasing expression with severity of disease. When examining expression of MMP-9 in intima, a linear trend was observed between disease severity and MMP-9 levels. On the other hand, expression of MMP-9 in subintima had parabolic trend with the peak level of 3301 ± 101.6 positive cells/mm^2^ in mild OA (Figure 3).

iNOS expression in both intima and subintima was positively correlated with Krenn score in moderate and severe OA groups. Expression of BCL-2 in intima of severe OA patients positively correlated with Krenn score. However, expression of BCL-2 in the subintima and expression of MMP-9 in both intima and subintima showed no correlation with Krenn score (Table 2).

**Figure 4:**
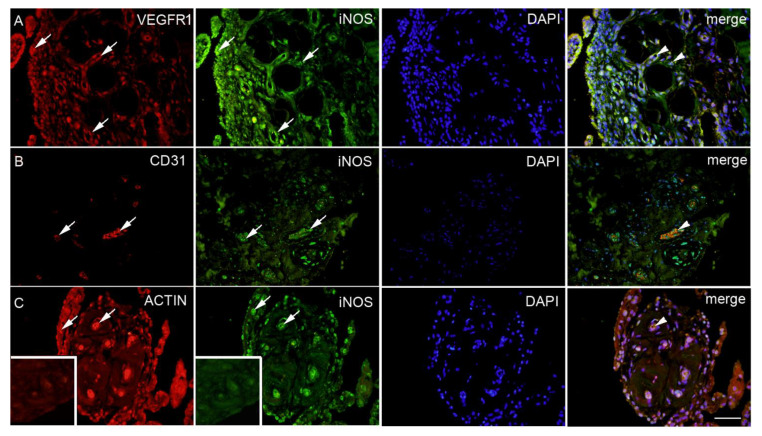
Synovial membrane of patients with hip osteoarthritis (HOA). VEGFR1 (vascular endothelial growth factor receptor 1) positive cells (red) in the synovial intima and subintima (first left arrow) and the blood vessels (arrows); iNOS positive cells (green) in the blood vessels and surrounding synovial intima and subintima (arrows); 4′,6-diamidino-2-phenylindole (DAPI) (blue) nuclear staining. Co-localization of VEGFR1 and iNOS merged with DAPI nuclear staining (arrowheads) is displayed in the far-right column (merge); moderate HOA (**A**). CD31 positive cells (red) in the blood vessels (arrows); iNOS positive cells (green) in the blood vessels within surrounding synovial subintima and superficial intima (arrows); DAPI (blue) nuclear staining; co-localization of CD31 and iNOS merged with DAPI nuclear staining (arrowheads) is displayed in the far-right column (merge); severe HOA (**B**). ACTIN smooth muscle (red) positive cells in in synovial intima and blood vessels (arrows); iNOS (green) positive cells in the blood vessels, synovial intima (arrows) and surrounding synovial subintima; DAPI (blue) nuclear staining; co-localization of ACTIN and iNOS merged with DAPI nuclear staining is displayed in the intima and blood vessels (arrowheads) in the far right column (merge); severe HOA (**C**). Inset: negative control of the synovial membrane. Magnification ×40, scale bar = 80 µm.

**Figure 5:**
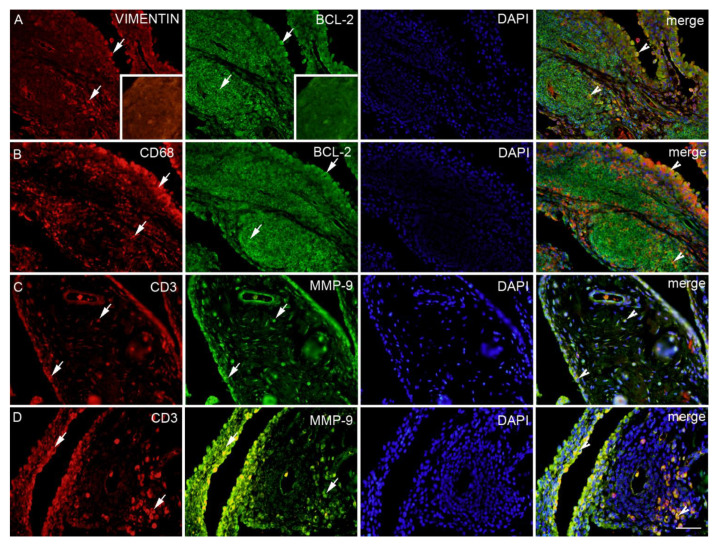
Synovial membrane of patients with hip osteoarthritis (HOA). Vimentin (red) fibroblasts and BCL-2 (green) positive cells in the intima and subintima (arrows); DAPI (blue) nuclear staining; co-localization of VIMENTIN and BCL-2 merged with DAPI nuclear staining is displayed in the intima and lymph node (arrowheads) in the far-right column (merge); severe HOA (**A**). CD68 (red) macrophages and BCL-2 (green) positive cells in the intima and subintima (arrows); DAPI (blue) nuclear staining; Co-localization of CD68 and BCL-2 merged with DAPI nuclear staining are displayed in the intima and lymph node (arrowheads) in the far-right column (merge); severe HOA (**B**). CD3 (red) and MMP-9 (green) positive cells in the intima and subintima (arrows); DAPI (blue) nuclear staining; co-localization of CD3 and MMP-9 merged with DAPI nuclear staining is displayed in the intima and subintima (arrowheads) in the far-right column (merge) in the moderate HOA (**C**). CD3 (red) and MMP-9 (green) positive cells in the intima and subintima (arrows); DAPI (blue) nuclear staining; co-localization of CD3 and MMP-9 merged with DAPI nuclear staining is displayed in the intima and lymph node (arrowheads) in the far-right column (merge) in the severe HOA (**D**). Inset: negative control of the lymph node. Magnification ×40, scale bar = 80 µm.

According to staining intensity to specific antibodies in moderate OA, severe OA and control, we observed the strongest staining intensity in of iNOS in moderate OA, BCL-2 in severe OA and MMP-9 in both moderate and severe OA (Table 3, Figure 4 and Figure 5). All of these markers co-localized with tissue specific cells (i.e., angiogenic cells, fibroblasts, macrophages and T-lymphocytes) (Figure 4 and Figure 5).

**Figure 6:**
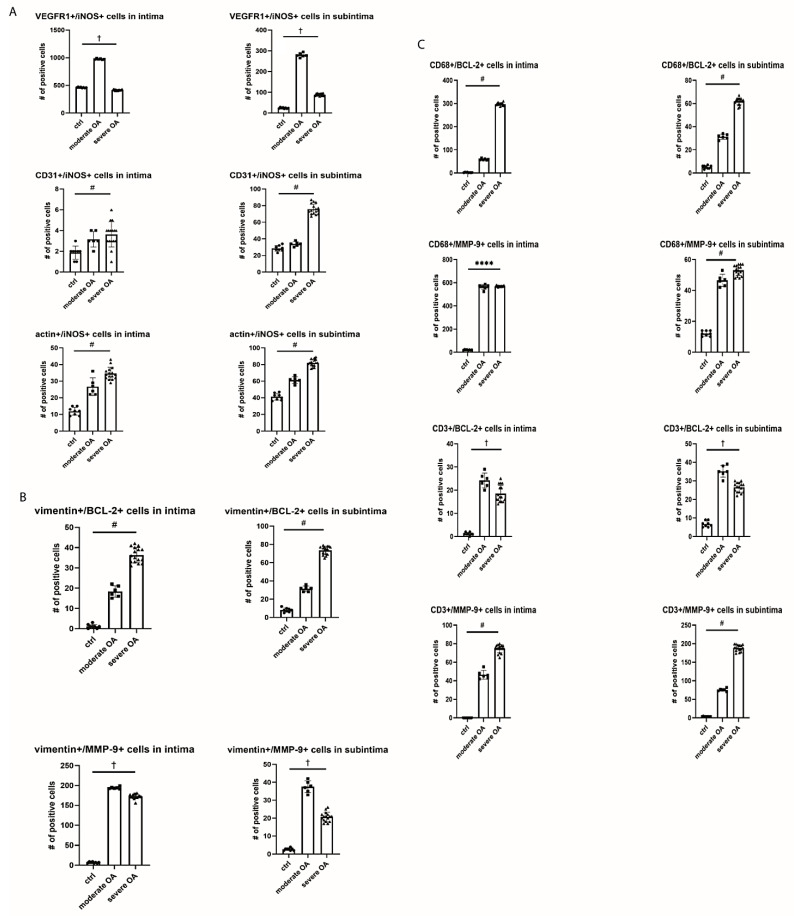
Expression of VEGFR1, CD31, BCL2 and MMP-9 in vascular cells (**A**), fibroblast like cells (**B**) and macrophages and T cells (**C**) in synovial tissue of OA patients. Legend: osteoarthritis (OA); ****, *p* < 0.0001 for ANOVA; #, *p* < 0.05 for the test for linear trend; † *p* < 0.05 for the test for parabolic (quadratic) trend.

In order to study potential angiogenesis in synovial tissue we observed numbers of cells which co-localized iNOS, BCL-2 and MMP-9 with different angiogenic markers (Figure 4). VEGFR1+/iNOS+ cells in intima and subintima showed a parabolic trend with peak expression of 975.3 ± 10.27 and 279.3 ± 10.01 positive cells/mm^2^, respectively, in moderate OA (Figure 6). We need to point out that the level of VEGFR1+/iNOS+ expression in subintima was almost 12 times higher in moderate OA than in control. Numbers of CD31+/iNOS+ as well as actin+/iNOS+ cells in both intima and subintima had linear trend with severity of disease. The highest level of CD31+/iNOS+ cells was observed in severe OA (75.75 ± 6.2 positive cells/mm^2^).

In order to examine fibroblast-like cells biology in context of antiapoptotic activity and EMC remodeling in both intima and subintima, we calculated the numbers of vimentin+/BCL-2+ cells that had a linear trend, while vimentin+/MMP-9+ had a parabolic trend (i.e., with the peak of 194 ± 2.82 in the moderate OA) with severity of disease. On the other hand, considering possible macrophages (i.e., CD68+ cells) in the same contexts, it was obvious that CD68+ cells were more prominent in antiapoptotic process (especially in severe OA intima), than in the EMC remodeling in both intima and subintima. Similarly, considering possible T-lymphocytes (i.e., CD3+ cells), which co-express BCL-2, there is parabolic trend in subintima, while T-lymphocytes that co-express MMP-9 showed linear increase with disease severity (Figure 6).

## 3. Discussion

OA has elements of inflammatory events, which are triggered by macrophages from the synovium. Studies of the knee OA have shown correlation of synovial inflammation and progression of disease [29] and correlation of synovial inflammation and sensation of pain expressed in a visual–analogue scale as well [30]. Macrophages and FLS from the synovium also secrete MMPs [31]. Given the important proinflammatory role of the synovial macrophages, FLS and T cells [32] in the pathogenesis of OA, in this paper we decided to investigate their co-localization with BCL-2, MMP-9 and iNOS in order to achieve a better insight into the inflammatory changes of the synovial membrane in HOA. Our results point to inflammation, remodeling and survival process in the synovial membrane of HOA patients. By analyzing BCL-2 (survival of inflammatory tissue), iNOS (important proinflammatory and an angiogenic molecule) and MMP-9 (tissue degradation and angiogenesis) in hip synovial membrane, we wanted to establish a correlation between their expression with histological changes of hip synovium during HOA. We found no differences between moderate and severe HOA groups according to Krenn score and K-L grade in HHS, WOMAC and VAS. HHS and WOMAC assess range of motion, difficulties in activities of daily living and pain. Activities of daily living are influenced by both, joint stiffness and pain. Therefore, it can be speculated that Krenn score does not influence on joint stiffness or sensation of pain. Since those scores are dependent on several components it is difficult to conclude whether Krenn score has influence on some of the HHS and WOMAC components. We found no correlation to VAS either. In the later stages of HOA, joint stiffness is probably caused by marked osteophytosis and joint capsule contraction, while, in moderate OA, decrease in HHS and WOMAC compared to healthy individuals may be caused by pain due to synovitis.

Our work showed that the numbers of synovial lining cell layers, resident cells in stroma and inflammatory infiltrate were increasing along with the severity of HOA according to Krenn score and K-L grade, probably due to more chronic inflammation. The limitation of this study is that we did not have an early OA group since specimens were taken during joint replacement surgery. Previous studies confirmed the existence of lymphoid infiltrate in OA synovia. In the study of knee OA, it was found in five out of twenty synovial membranes [33], while in the study investigating hip and knee OA, CD3+ aggregates were found in 65% of synovial membranes [34]. We found one quarter of synovial membranes in the severe OA group that showed the presence of lymphoid aggregates. Lymphoid aggregates are present only in advanced OA. Results may vary because of the different influences from surrounding tissues i.e., synovial membrane in the knee may be influenced by the infrapatellar fat pad as a source of pro-inflammatory cytokines [35]. T cells are the major represented cells in OA lymphoid infiltrate [36] while they represent about 22% of the immune cells that the infiltrate OA synovial membrane. Macrophages are the most represented immune cell of the inflamed synovial membrane in OA and make about 65% of infiltrate [37]. Those studies were conducted on the knee OA synovial membrane. Our study confirms that the most abundant cells in hip OA synovia were presumably macrophages according to the high number of CD68+/MMP-9+ and CD68+/BCL-2+ cells. However, although using surface marker to distinguish cell type is common, clarifying the cell type with only marker is risky and, therefore, these results should be carefully interpreted. Additionally, another limitation is that this study is an observational study and did not include the functional role of each marker. Therefore, the changes of these markers may not play a dominant role in the progression of OA.

The inflammation process is influenced by iNOS [38]. Ostojic et al. reported higher expression of iNOS in an early radiological knee OA then in advanced OA [24]. Although we did not have an early OA group, our results were in line with that, considering that moderate OA had higher levels of iNOS, but a lower synovitis score than severe. It is possible that higher levels of iNOS expression precede a higher Krenn score in the severe OA group. Additionally, positive correlation with Krenn score is established between the control and both HOA groups. Krenn score is based on enlargement of lining cell layers, cellular density of synovial stroma and leukocytic infiltrate [39]. BCL-2 expression was increased in both HOA groups with a linear trend in the severe HOA group in the subintima, emphasizing the importance of the antiapoptotic mechanism in the synovial inflammatory cells and fibroblast-like synoviocytes that contribute to inflammation. In line with the fact that inflammation process is influenced by iNOS is our observation of the most prominent co-localization of iNOS and VEGFR1 positive cells, especially in the subintima of moderate OA patients. Vascular endothelial growth factor (VEGF) is produced by inflamed synovium during OA and may promote angiogenesis [40]. Angiogenesis may be present in all stages of OA and is associated with chronic synovitis [41]. Contrarily, severe OA has shown more co-localization of iNOS with maturated blood vessels i.e., CD31 and actin positive cells.

Based on vimentin+/MMP-9 co-localization among the groups it seems that matrix remodeling is most intensive in moderate OA, while the survival ability of fibroblast-like cells increases with severity of OA. Increased BCL-2 expression in the synovial fibroblasts is documented in the RA (rheumatoid arthritis) synovial membrane where it contributes to inflammation [42] and is more pronounced than in OA synovial fibroblasts. Little is known about the same process in OA. We observed a similar but less intense process in HOA. Fibroblasts protection from apoptosis may contribute to synovial inflammation.

Considering possible macrophages in the same contexts, they were more prominent in severe OA intima, implying their long life potential. They showed strong co-expression with MMP-9 in both HOA groups which was anticipated given their role in OA pathogenesis [9]. T-lymphocytes have their helper role in the subintima of moderate OA while co-localizing BCL-2 and contributing to the remodeling process in severe OA.

In conclusion, matrix remodeling and angiogenesis might play more a prominent role in moderate OA, whereas severe OA seems to be marked by fibrosis and completed angiogenesis; the latter is accompanied by increased long living macrophages infiltration.

## 4. Materials and Methods

### 4.1. Study Population

This cross-sectional study was approved by The Ethics Committee of the University Hospital in Split in accordance with the 1964 Helsinki declaration. Thirty-two patients were included in this study. There were three groups of patients: displaced femoral neck fracture in ambulatory patients older than 65 years (control group, No 8), moderate osteoarthritis group (No 6), and severe osteoarthritis group (No 18). In the OA group only patients with a diagnosis of primary HOA were included. Exclusion criteria for HOA groups were hip dysplasia, history of hip fracture or infection and history of rheumatic conditions. Osteoarthritis groups were scored according to the Harris Hip Score (HHS), Western Ontario and McMaster Universities Arthritis Index (WOMAC), visual analogue scale (VAS) and the radiologic Kellgren–Lawrence (K-L) grading scale. HHS, WOMAC and VAS were obtained for osteoarthritis groups within 1 month before surgery by the same orthopedic surgeon. The HHS and WOMAC and were not assessed in patients with a femoral neck fracture since these two scores assess the range of motion. VAS was obtained only for OA groups. In order to be put in the group of severe OA patients had to reach a K-L grade of at least 3, and a Krenn score at least 7. The patients used as control all had K-L grades of 0 or 1. Standard anteroposterior hip radiographs for OA group were taken no more than 6 months prior to a surgery. HOA diagnosis was established according to the American College of Rheumatology criteria for the classification and reporting of osteoarthritis of the hip [43]. Indication for surgery in HOA patients was failed conservative treatment with persistence of pain and functional limitations. The written informed consent form was signed by each patient prior to surgery, after the procedure was explained by an orthopedic surgeon. Age, gender, body mass index (BMI), symptoms duration, and the radiological stage of the disease were documented.

### 4.2. Tissue Collection and Basic Staining Procedures

All patients underwent total hip arthroplasty (Pinnacle Acetabular Cup System and Corail Hip System by Depuy, Warsaw, IN, USA) at the Department of Orthopaedics and Traumatology of University Hospital in Split. Total hip arthroplasty was performed through posterolateral hip approach with posterior capsule incision. Tendons of m. piriformis, m. obturator internus and both mm. gemellis were divided. After luxation of the hip, femoral neck was osteotomized by a 1.3 mm saw (Trauma Reckon System by Synthes, Switzerland) and femoral head together with a part of femoral neck was extracted. In trauma cases there was no need for hip luxation, but femoral head was removed by a corkscrew after femoral neck osteotomy followed by partial femoral neck extraction. Then, a sample from the weight bearing area of the femoral head was taken by a sew in triangular manner. Samples were taken adjacent to the most damaged part of the weight bearing area but still containing a cartilage. Synovial tissue from inferior part of the femoral neck adjacent to the femoral head was taken.

The tissue samples were collected in the period of 2017 to 2020. Samples were put into formalin solution and sent to the Department of Anatomy, Histology and Embryology of the University of Split School of Medicine for histological analysis. Cartilage and subchondral degeneration was assessed using the OARSI grading system, while the synovial membrane was assessed according to Krenn score with 0 to 1 corresponding to no synovitis (inflammatory grade = 0), 2 to 3 to a slight synovitis (inflammatory grade 1), 4 to 6 to a moderate synovitis (inflammatory grade 2), and 7 to 9 to a strong synovitis (inflammatory grade 3). Tissues were formalin fixed, decalcified with 14% ethylenediaminetetraacetic acid (EDTA) solution (four months), paraffin embedded and cut to 5-μm thick sections. Each 10th section was stained with hematoxylin and eosin.

### 4.3. Double Immunofluorescence

After deparaffinization, sections for immunohistochemistry were rehydrated in decreased ethanol grades as we described previously [44,45,46,47]. Briefly, the slides were heated in citrate buffer (pH 6.0) in a microwave oven for 12 min. After cooling off at room temperature the slides were rinsed with Phosphate-Buffered Saline (PBS). Block protein was then put on the tissue sections for 30 min following overnight incubation with appropriate primary antibodies mixture (Table 4). On the next day, the slides were rinsed with PBS and an appropriate combination of secondary antibodies was applied (Table 4). The nuclei were stained with 4′,6-diamidino-2-phenylindole (DAPI) and slides were mounted in Immu-mont and cover slipped. Slides were examined using an Olympus (Tokyo, Japan) BX51 microscope equipped with a Nikon DS-Ri1 camera (Nikon Corporation, Tokyo, Japan). Images were assembled using Adobe Photoshop (Adobe Systems, MI, USA). Ten non overlapping fields were taken using 40× objective magnification. Double immunofluorescence with primary antibodies to iNOS, BCL-2 and MMP-9 was used in a combination of different specific cell type markers (Table 4), to determine the number of positive cells in the surface layer of cells (intima) and the underlying tissue (subintima) of the synovial membrane. We counted only cells that displayed both markers in the same cell (red or green signal) in the nucleus or cytoplasm. The cell count was performed using ImageJ software (National Institutes of Health, Bethesda, MD, USA). The total number of VEGFR1+/iNOS+, CD31+/iNOS+, actin+/iNOS+, vimentin+/BCL-2+, CD68+/BCL-2+, CD3+/BCL-2+, vimentin+/MMP-9+, CD68+/MMP-9+, CD3+/MMP-9+ positive cells were calculated as the number of cells per mm2 in the intima and subintima of the synovial membrane. The final total number per patient was the mean of 20 sections that were calculated and compared between groups (control, moderate and severe OA).

### 4.4. Statistical Analysis

Data analysis was conducted with GraphPad Prism (GraphPad Software, La Jolla, CA, USA). Nominal variables are presented as fraction, ordinal and continuous variables are presented as the median and interquartile range or mean and standard deviation. To test for differences and trends between groups ANOVA, a Kruskal–Wallace test and tests for linear or parabolic (quadratic) trends were used. For modelling relationships between different variables, linear regression was used. Statistical evidence is presented as R2 and *p* values. *p* values were interpreted according to the American Statistical Association Statement on *p* values.

## Figures and Tables

**Table 1:** Clinical, radiological and pathohistological characteristics of the examined groups.

	Controls	Moderate OA	Severe OA	* *p* Value
Age (median ± IQR, years)	73 (72.25–76.75)	72 (63.5–75.5)	72 (67–77)	0.884
BMI (median ± IQR, kg/m^2^)	25.67 (23.83–26.8)	24.9 (23.28–25.88)	26.9 (25.4–29.53)	0.055
K-L grade (median ± IQR)	0.5 (0–1)	2 (2–2)	4 (3–4)	˂0.0001
Krenn score (median ± IQR)	0 (0–0)	6.5 (5.7–9)	9 (7–9)	˂0.0001
OARSI (median ± IQR)	1 (0.25–1)	3 (1.5–4.5)	2.5 (2–4.7)	0.0005
HHS (median ± IQR)	–	48.9 (43.8–56.9)	41 (33.18–49.7)	0.271
VAS (median ± IQR)	–	6 (4.5–6.7)	6 (5–7)	0.781
Total WOMAC (median ± IQR)	–	46.1 (40–57.4)	47.3 (36.1–55.3)	0.917

IQR (interquartile range), OA (osteoarthritis), BMI (body mass index), K-L grade (Kellgren–Lawrence grading scale), OARSI (osteoarthritis cartilage histopathology assessment system), HHS (Harris Hip Score), VAS (visual analogue scale), WOMAC (The Western Ontario and McMaster Universities Osteoarthritis Index) * *p* < 0.05, Kruskal–Wallace test.

**Table 2:** Correlation of disease severity (β) and Krenn score (α) with expression of iNOS, BCL-2 and MMP-9 in intima and subintima of OA patients.

	Disease Severity								
	Control			Moderate OA			Severe OA		
	α	β	R^2^	α	β	R^2^	α	β	R^2^
Age (years)									
iNOS intima	0	727.3 (692.9–761.6)	0	556.6 (446.2–667)	3003 (2212–3793)	98% *	372.7 (319.3–426.1)	670.2 (220–1120)	94% *
iNOS subintima	0	59 (49.3–68.7)	0	supplementary Figure S1	7464 (7023–7904)	99% *	67.45 (53.85–81.04)	–154.9 (–269.5–0)	89% *
BCL-2 intima	0	3110 (2769–3451)	0	0	3504 (3108–3900)	0	177.2 (56.26–298.1)	2022 (1003–3041)	41% *
BCL-2 subintima	0	4134 (3845–4423)	0	0	4460 (4230–4689)	0	0	7194 (6762–7626)	0
MMP-9 intima	0	261 (234–288)	0	0	7223 (7043–7404)	0	0	7465 (7292–7638)	0
MMP-9 subintima	0	33.88 (23.42–44.33)	0	0	3252 (2652–3852)	0	0	2184 (1304–3065)	0

* ANOVA *p* value for model < 0.0001; α–slope; β–intercept. Data in the brackets indicate 95% CI of model parameter. Number of examined iNOS, BCL-2 and MMP-9 positive cells are expressed as number of cells/mm^2^.

**Table 3:** Staining intensity to specific antibodies in moderate OA, severe OA and control.

Antibodies	Diagnosis		
	Moderate OA	Severe OA	Control
iNOS	+++	++	+
BCL-2	++	+++	++
MMP-9	+++	+++	+

Three pluses indicate strong reactivity; two pluses indicate moderate reactivity; one plus indicates mild reactivity; minus indicates no reactivity.

**Table 4:** Primary and secondary antibodies used.

Antibodies	Host	Dilution	Structures Identified by Antibodies	Source
ab59348 (polyclonal antibody)	Rabbit	1:500	BCL-2	Abcam (UK)
sc-651 (monoclonal antibody)	Rabbit	1:200	iNOS	Santacruz Biotechnology (Santa Cruz, CA, USA)
A0150 (polyclonal antibody)	Rabbit	1:100	MMP-9	DAKO (Gloustrup, Denmark)
M0823 (monoclonal antibody)	Mouse	1:20	CD31 (endothelial cells of blood vessels)	DAKO (Gloustrup, Denmark)
M0851 (monoclonal antibody)	Mouse	1:40	Actin (smooth muscle cells of blood vessels)	DAKO (Gloustrup, Denmark)
ab212369 (monoclonal antibody)	Mouse		VEGFR1	Abcam (UK)
M0725 (monoclonal antibody)	Mouse	1:50	Vimentin (fibroblasts)	DAKO (Gloustrup, Denmark)
M0876 (monoclonal antibody)	Mouse	1:75	CD68 (macrophages)	DAKO (Gloustrup, Denmark)
M7254 (monoclonal antibody)	Mouse	1:50	CD3 (lymphocytes)	DAKO (Gloustrup, Denmark)
Rhodamine Goat AP124R	Mouse	1:100	Secondary antibody	MerckMillipore (Billerica, MA, USA)
Fluorescein Goat AP132F	Rabbit	1:100	Secondary antibody	MerckMillipore (Billerica, MA, USA)

## Data Availability

The data presented in this study are available on request from the corresponding author.

## Supplementary Materials

The following are available online at https://www.mdpi.com/1422-0067/22/3/1489/s1, Figure S1: Relationship between Krenn score and iNOS expression in subintima of OA patients.
