# Supplementary material for: Expression Pattern of iNOS, BCL-2 and MMP-9 in the Hip Synovium Tissue of Patients with Osteoarthritis
Source: Int J Mol Sci. 2021 Feb 2;22(3):1489. doi: 10.3390/ijms22031489 (PMC7867378; doi:10.3390/ijms22031489)
Supplement: Supplementary file 1 [file ijms-22-01489-s001.pdf]

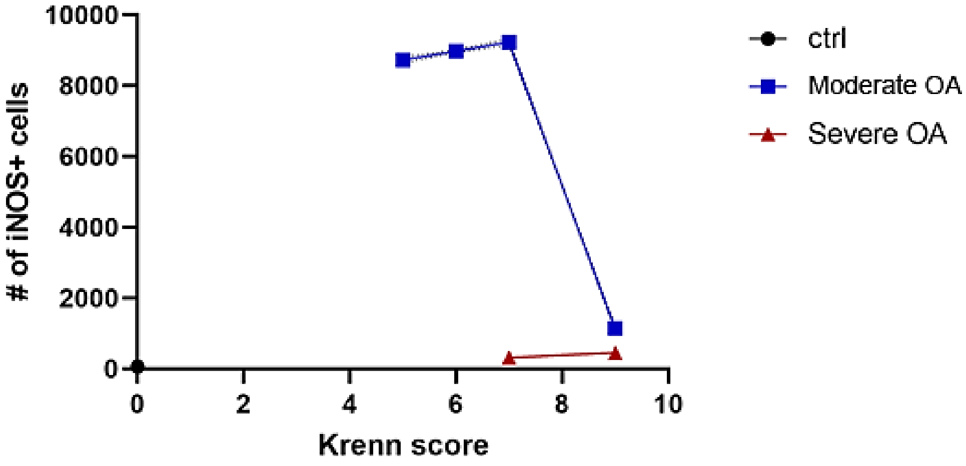

Figure S1. Relationship between Krenn score and iNOS expression in subintima of OA patients. Patients with moderate OA demonstrate a bi-segmental linear relationship between iNOS expression and Krenn score (β= 7464, 95% CI: 7023 to 7904, R^2 = 100%, p < 0.0001). For scores up to 7 positive correlation between iNOS levels and Krenn score exsists (α_1=252.2, 95%CI: 179.6 to 325.4,), whereas for scores grater then 7 the relationship is negative (α_2= −4044, 95%CI: −4102 to −3987). Legend: control (ctrl) osteoarthritis (OA); α_1, slope parameter before changepoint in bi-segmental linear model, α_2 slope parameter after the changepoint in bi-segmental linear model, β, intercept.
